# Germany has a high demand in meniscal allograft transplantation but is subject to health economic and legal challenges: a survey of the German Knee Society

**DOI:** 10.1007/s00167-022-06889-5

**Published:** 2022-01-31

**Authors:** Philipp W. Winkler, Svea Faber, Maurice Balke, Sebastian Metzlaff, Thomas R. Niethammer, Philip P. Roessler, Ralf Henkelmann, Alexander Kurme, Sebastian Colcuc, Gerald Zimmermann, Wolf Petersen, Theresa A. Diermeier

**Affiliations:** 1 Department for Orthopaedic Sports Medicine, Klinikum rechts der isar, Technical University of Munich, Ismaninger Str. 22, 81675 Munich, Germany; 2 OCM Orthopädische Chirurgie München, Munich, Germany; 3 Sportsclinic Cologne, Universität Witten/Herdecke, Köln, Germany; 4 St. Joseph Krankenhaus, Berlin, Germany; 5 Department of Orthopaedics and Trauma Surgery, Musculoskeletal University Center Munich (MUM), University Hospital, LMU Munich, Munich, Germany; 6 Gelenkzentrum Mittelrhein, Koblenz, Germany; 7 Department of Orthopedics, Trauma and Plastic Surgery, University of Leipzig, Leipzig, Germany; 8 ATOS Klinik Fleetinsel Hamburg, Hamburg, Germany; 9 Klinik für Arthroskopische Chirurgie, Sporttraumatologie und Sportmedizin, BG Klinikum Duisburg,, Duisburg, Germany; 10 Unfallchirurgie und Sporttraumatologie, TheresienkrankenhausMannheim, Mannheim, Germany; 11 Sportklinik Berlin und Klinik für Orthopädie und Unfallchirurgie, Martin-Luther-Krankenhaus, Berlin, Germany; 12 Unfallkrankenhaus Berlin, Berlin, Germany

**Keywords:** Meniscus deficiency, Post-meniscectomy syndrome, Allograft, Reconstructive surgery, Biologic knee reconstruction, Fixation technique

## Abstract

**Purpose:**

To determine the current status and demand of meniscal allograft transplantation (MAT) in Germany among members of the German Knee Society (= Deutsche Kniegesellschaft; DKG).

**Methods:**

An online survey was conducted between May 2021 and June 2021 and sent to all members of the DKG. The survey questionnaire consisted of 19 questions to determine the demand and technical aspects of MAT among the participants and to identify areas of improvement in MAT in Germany.

**Results:**

Overall, 152 participants, 136 (89.5%) from Germany, 8 (5.3%) from Switzerland, 6 (4.0%) from Austria, and 2 (1.3%) from other countries completed the online survey, with the majority working in non-academic institutions. According to the regulations of the DKG, 87 (57.2%) participants were board certified as specialized knee surgeons and 97 (63.8%) worked primarily in the field of orthopedic sports medicine. MAT was considered clinically necessary in Germany by 139 (91.5%) participants. Patient age (83.6%), post-meniscectomy syndrome in isolated lateral (79.6%) and medial (71.7%) meniscus deficiency, and functional and athletic demands (43.4%) were the most important determinants to consider MAT in patients. Participants reported that reimbursement (82.9%), jurisdiction over the use of donor grafts (77.6%), and the availability of meniscal allografts (76.3%) are the main challenges in performing MAT in Germany. The most frequently used meniscal allograft types by 54 (35.5%) participants who had already performed MAT were fresh-frozen grafts (56.6%), peracetic acid–ethanol sterilized grafts (35.9%), and cryopreserved grafts (7.6%). Participants reported to perform suture-only fixation more often than bone block fixation for both medial (73.6% vs. 22.6%) and lateral (69.8% vs. 24.5%) MAT.

**Conclusion:**

More than 90% of the responding members of the DKG indicated that MAT is a clinically important and valuable procedure in Germany. Reimbursement, jurisdiction over the use of donor grafts, and the availability of meniscal allografts should be improved. This survey is intended to support future efforts to facilitate MAT in daily clinical practice in Germany.

**Level of evidence:**

Level V.

**Supplementary Information:**

The online version contains supplementary material available at 10.1007/s00167-022-06889-5.

## Introduction

Given the high-quality evidence that meniscal resection inevitably affects knee biomechanics and results in early onset osteoarthritis (OA) [25], there is no doubt that meniscus-preserving procedures should be advocated. Despite major advances in meniscus-preserving technologies over the past years, a recent study showed that 65% of arthroscopically evaluated meniscal tears were still considered irreparable [7]. In such cases, partial, subtotal, or even total meniscus resection remains the only treatment option. A subgroup of patients who have undergone subtotal/total meniscal resection are likely to develop symptomatic meniscus deficiency or even post-meniscectomy syndrome. Stabbing joint line pain and recurrent effusions affect athletic performance and reduce the quality of life [5]. Meniscal allograft transplantation (MAT) has been shown to be a viable option to treat patients with symptomatic meniscus deficiency and thereby improve symptoms, athletic performance, and the quality of life [2, 4, 5, 24].

However, the availability of meniscal allografts varies immensely among countries around the world. German jurisdiction of donor graft procurement, preservation, and transplantation, as well as reimbursement, present major challenges that can considerably delay or even prevent timely MAT [19]. The German Knee Society (= Deutsche Kniegesellschaft; DKG) was founded in 2012 as a group of experts in the field of arthroplasty, traumatology, sports medicine, and rehabilitation of the knee joint. The DKG includes physicians, medical students, and physical therapists. Given the limited availability of meniscal allografts in Germany, a subgroup of DKG members felt compelled to investigate the current status and demand of MAT in Germany. A profound investigation of the demand for and limitations of MAT in Germany should support future efforts to implement MAT in daily clinical practice in Germany.

The objective of this study was to determine the current status and demand of MAT in Germany among members of the DKG. It was hypothesized that the availability and clinical use of meniscal allografts in Germany is limited, although great demand exists.

## Materials and methods

This study was approved by the board of the DKG prior to data collection. An online survey on MAT was conducted and sent to all members of the DKG between May 2021 and June 2021. Participation in the survey was voluntary. Refusal to participate in the survey had no adverse consequences for members. The survey consisted of 19 questions and was distributed via an online survey provider (SurveyMonkey Inc., San Mateo, CA, USA). Each data entry was processed anonymously and the survey was accessible for a period of 2 months. A subgroup of 4 orthopedic surgeons (PWW, SF, TAD, and WP) from the DKG “Cartilage and Meniscus” committee developed the survey questions based on a preceding interactive discussion with all members of the committee. The survey questionnaire was designed to determine the demand and technical aspects of MAT among the participants and to identify areas of improvement in MAT in Germany (Supplement 1).

Since November 2014, the DKG has offered the opportunity to apply for certification as a specialized knee surgeon. To be board certified as a specialized knee surgeon, the following criteria must be met: (1) Completed 6-year residency specializing in orthopedic surgery and traumatology, (2) completed curriculum consisting of theoretical and practical trainings as defined by the board of the DKG, and (3) proof of at least 500 self-performed advanced-level knee surgical procedures as defined by the board of the DKG.

### Statistical analysis

After the 2-month period for data entry, all data were summarized and extracted from the online survey database. The number of respondents per answer and the corresponding percentage were reported. Since more than one answer could be selected in some questions, the cumulative percentage for these questions may exceed 100%.

## Results

The survey questionnaire and the corresponding results can be found in Supplement 1. In total, 152 participants, 136 (89.5%) from Germany, 8 (5.3%) from Switzerland, 6 (4.0%) from Austria, and 2 (1.3%) from other countries successfully completed the online survey. The majority of participants reported to work in non-academic institutions, while 24 (15.8%) worked at a university hospital. Of all respondents, 87 (57.2%) were board certified as specialized knee surgeons and 97 (63.8%) worked primarily in the field of orthopedic sports medicine.

More than 100 meniscal surgeries are performed annually by 83 (54.6%) participants and 139 (91.5%) participants indicated that MAT is clinically necessary in Germany.

Meniscal allograft transplantation is considered as a treatment option in at least 5 patients annually by 90 (59.2%) respondents.

The most important determinants to consider MAT in patients were patient age (127 votes, 83.6%), post-meniscectomy syndrome in isolated lateral (121 votes, 79.6%) and medial (109 votes, 71.7%) meniscus deficiency, and functional and athletic demands (66 votes, 43.4%).

Fifty-four (35.5%) participants have performed MAT, with 35 (64.2%), 7 (13.2%), and 12 (22.6%) of these participants reporting having performed < 5, 5–10, and > 10 MATs in their careers, respectively.

Meniscal allograft types used included fresh-frozen grafts (56.6%), peracetic acid–ethanol (PAA) sterilized grafts (35.9%), and cryopreserved grafts (7.6%).

Thirty (54.7%), 22 (41.5%), and 2 (3.8%) participants reported to perform MAT with an arthroscopic, arthroscopically assisted, and open technique, respectively.

Medial meniscal allograft root fixation is performed using the bone block and suture-only technique by 12 (22.6%) and 40 (73.6%) participants, respectively. For lateral meniscal allograft root fixation, the bone block fixation technique is used by 13 (24.5%) participants and the suture-only technique by 37 (69.8%) of participants.

**Fig. 1:**
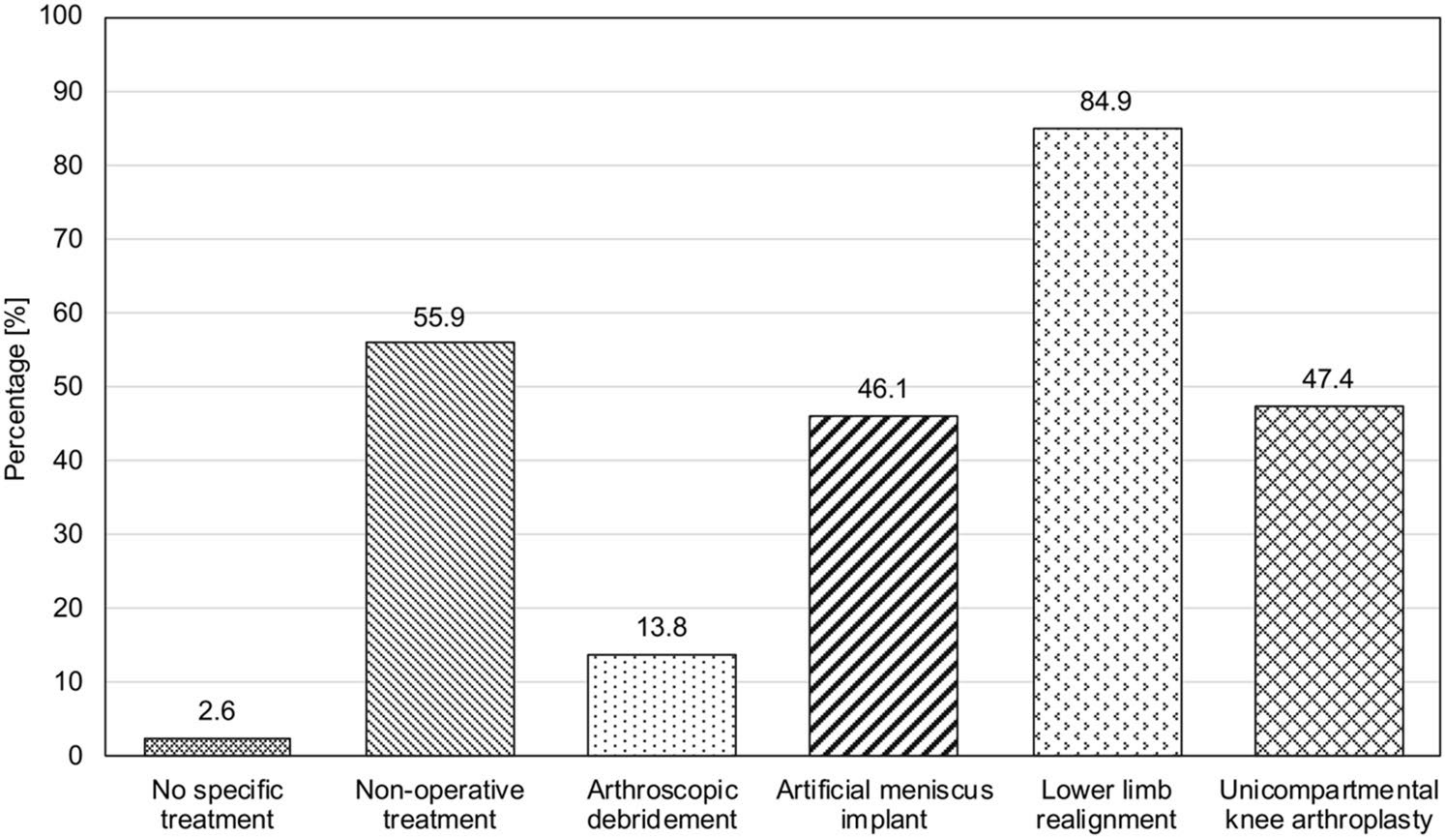
Treatment alternatives to meniscal allograft transplantation in Germany. The bars indicate the percentage of participants who voted for each answer. Since more than one answer could be selected, the cumulative percentage exceeds 100%

When MAT is not possible, the most popular treatment alternatives were operative lower limb realignment (129 votes, 84.9%), non-operative treatment (i.e., physical therapy; 85 votes, 55.9%), and the use of unicompartmental knee arthroplasty (UKA; 72 votes, 47.4%), and artificial meniscal implants (70 votes, 46.1%; Fig. 1).

**Fig. 2:**
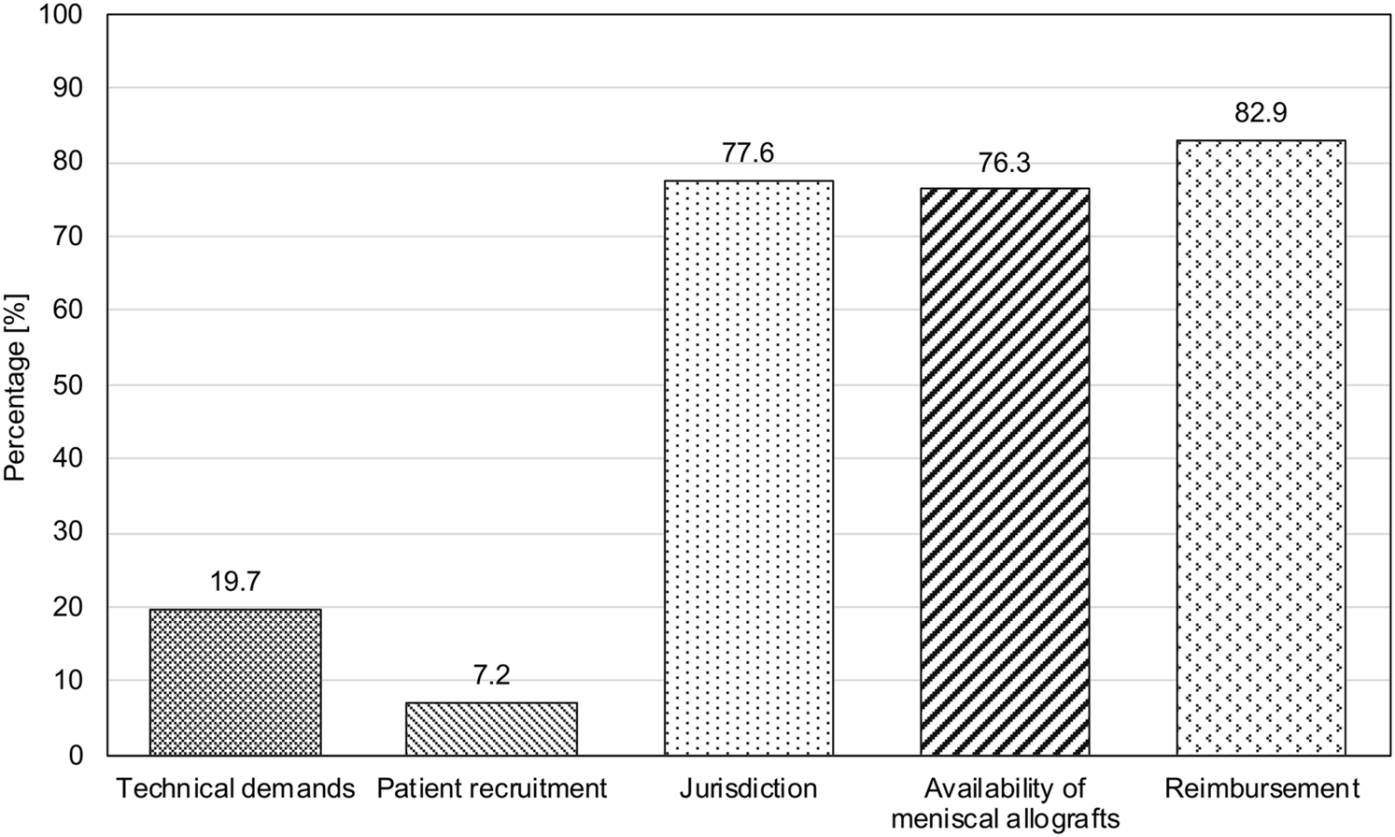
Challenges in meniscal allograft transplantation in Germany. The bars indicate the percentage of participants who voted for each answer. Since more than one answer could be selected, the cumulative percentage exceeds 100%

Reimbursement (82.9%), jurisdiction over the use of donor grafts (77.6%), and the availability of meniscal allografts (76.3%) were identified as the main challenges in performing MAT in Germany (Fig. 2).

## Discussion

The most important finding of this study was that more than 90% of the responding members of the DKG indicated that MAT is a clinically important and valuable procedure in the treatment of patients with symptomatic meniscus deficiency in Germany, but is subject to health economic and legal challenges.

Loss of meniscal tissue increases tibiofemoral contact pressure in the affected compartment and results in increased translational and rotatory knee laxity [1, 12, 16]. Persistent tibiofemoral overload causes degeneration of the articular cartilage, joint pain, loss of function, and ultimately early onset knee OA [11]. To mitigate such socioeconomic consequences for health care systems, meniscus- and joint-preserving treatment strategies have become increasingly important [14, 15]. For partial meniscus deficiency, artificial, scaffold-based meniscal substitutes have been proposed [25]. A prospective, multicenter study found significant improvements in patient-reported outcome scores in 137 patients undergoing partial meniscal replacement (Actifit®, Orteq Sports Medicine Ltd., London, United Kingdom) at 2 and 5 years after implantation [22]. However, reported mean defect sizes for scaffold-based meniscal substitutes range from 36 to 48 mm, requiring other treatment modalities for larger defects [25]. In such cases, MAT has emerged as a viable treatment option with high patient satisfaction. A recent study investigating 38 patients after MAT showed that 60–82% of patients still reached the patient acceptable symptom state 10 years after implantation [8]. High rates of return to sport further endorse MAT as the treatment of choice for symptomatic meniscus deficiency. A systematic review indicated a rate of return-to-play of 77% after a mean time of 9 months after MAT, with 68% of patients returning to the same or a higher level [9]. Although there is considerable evidence that MAT improves symptoms and athletic performance, there has only been one clinical trial comparing MAT to an alternative non-operative treatment option [23, 24]. In this pilot randomized clinical trial, patients with symptomatic meniscus deficiency undergoing MAT displayed significantly higher scores in the Knee Injury and Osteoarthritis Outcome Score (KOOS) subscales pain and activities of daily living than patients treated with personalized physiotherapy at the 12-month follow-up [20].

Given these persuasive results, it is not surprising that 91.5% of participants of this study indicated that MAT is a clinically important and valuable procedure in the treatment of patients with symptomatic meniscus deficiency in Germany. Meniscal allograft transplantation is a sophisticated surgical procedure that requires a certain level of surgical experience and comprehensive knowledge to provide the best patient care possible. Therefore, one might assume that MAT is required primarily by specialized academic hospitals. However, in this survey, only 15.8% of participants reported working in a university hospital, while 84.2% of participants reported working in other institutions such as peripheral hospitals or private practices. This reflects the importance of MAT for the entire German population and not just for individual cases. However, directing patients to approved centers could address some of the main challenges in performing MAT in Germany. In approved centers, the availability of meniscal allografts could be facilitated by the establishment of an in-house tissue bank. Moreover, continuous outcome analysis would be better controlled.

In Germany there are three laws (Medicinal Products Act, Transplantation Act, and Tissue Act) that regulate procurement, preservation, and transplantation of human donor tissue. Another important fact is that meniscus allografts in Germany are legally treated as medicinal products and not as organ transplants. The eligibility of each potential tissue donor is verified by a comprehensive screening protocol before donor tissue is harvested. As a result, the risk of graft contamination and disease transmission is minimized [18]. The clinical use of fresh-frozen and cryopreserved meniscal allografts is currently not approved in Germany, as each graft must undergo a sterilization process. Therefore, it is interesting that 56.6% of survey participants reported to use fresh-frozen meniscal allografts in MAT. Given that the German jurisdiction prohibits the manufacturing authorization of non-sterilized musculoskeletal donor tissue, fresh-frozen meniscal allografts cannot be legally manufactured in Germany. Yet, there is a possibility to import non-sterilized meniscal allografts (i.e., fresh-frozen allografts) from other countries of the European Union (EU) and from non-EU countries [18]. However, a considerable time and financial effort is required, since either an approval for importation for donor tissue by the responsible administration office is required or an application for exemption must be granted for each individual case [3]. Otherwise, the surgeon performing MAT may be subject to legal liability for complications related to the graft. Moreover, it should be noted that almost 11% of the participants stated that they do not practice in Germany and are subject to different legislation.

Although there are concerns that sterilization of musculoskeletal donor tissue adversely affects its biomechanical and biological properties [10], sterilization is required to obtain a manufacturing authorization for meniscal allografts in Germany. Therefore, PAA-sterilized meniscal allografts are popular in Germany, as shown by a proportion of 35.9% of the participants performing MAT in this survey. In a recent biomechanical study, 13 pairs of PAA-sterilized and fresh-frozen meniscal allografts, respectively, were compared and no difference in stiffness (14.9 N/mm vs. 18.3 N/mm) and load to failure (50.5 N vs. 59.5 N) testing was observed [6]. The authors suggested that the observed significantly higher strain (18.9% vs. 13.8%) and lower relaxation (77.7% vs. 89.1%) and moisture content in PAA-sterilized compared to fresh-frozen meniscal allografts may positively affect postoperative graft extrusion [6]. Despite an ongoing prospective multicenter study on the use of PAA-sterilized meniscal allografts, no clinical data are currently available. Other countries, such as the United States, allow the use of non-sterilized meniscal allografts. The risk of disease transmission is mitigated by thorough donor screening and testing, and strict regulations during tissue procurement and processing [10].

In this study, more than 50% of participants stated that they would perform MAT in 5–20 patients per year, which corresponds to approximately 0.5–1 MAT per month. The main determinants for indication of MAT reported by the participants were patient age (127 votes, 83.6%), post-meniscectomy syndrome in isolated lateral (121 votes, 79.6%) and medial (109 votes, 71.7%) meniscus deficiency, and functional and athletic demands (66 votes, 43.4%), which is consistent with the previous research [17]. Given the difficulties in performing MAT in Germany, the most popular treatment alternatives according to the survey were operative lower limb realignment (129 votes, 84.9%), non-operative treatment (85 votes, 55.9%), and the use of UKA (72 votes, 47.4%), and artificial meniscal implants (70 votes, 46.1%). Non-operative treatment of post-meniscectomy syndrome is sometimes recommended as first-line therapy, yet success rates and existing evidence are sparse [5]. Favorable short-, medium-, and long-term outcomes after lower limb realignment procedures have been consistently reported, with survival rates of up to 85% at 20 years [13, 21]. However, in patients with physiologic joint orientation angles, other treatment options such as MAT are warranted, since osteotomies may cause joint line obliquity. Furthermore, young patient age and high athletic expectations should question the indication for lower limb realignment and UKA in patients.

Although surveys provide valuable results from a particular group of interest, there are some limitations that have to be acknowledged. One limitation of this study is that only approximately 20% of the members of the DKG participated in the survey. However, 57.2% of participants were board certified as specialized knee surgeons and more than 50% of participants reported to perform more than 100 meniscus surgeries annually, representing a highly experienced cohort of experts in meniscus surgery. This study represents an expert opinion on the current status and demand of MAT in Germany rather than objective evidence. However, this survey is intended to support future efforts to facilitate MAT in daily clinical practice in Germany. Future studies should focus on objective data from national health care databases to further emphasize the importance of MAT in Germany and its associated limitations.

## Conclusions

In Germany, MAT is a clinically important and valuable procedure in the treatment of patients with symptomatic meniscus deficiency, yet its clinical implementation is subject to health economic and legal challenges. Reimbursement, jurisdiction over the use of donor grafts, and the availability of meniscal allografts should be improved to make MAT a clinically attractive therapy in Germany.

## Supplementary Information

Below is the link to the electronic supplementary material.

Supplementary file1 (DOCX 25 KB)
