# Supplementary material for: Germany has a high demand in meniscal allograft transplantation but is subject to health economic and legal challenges: a survey of the German Knee Society
Source: Knee Surg Sports Traumatol Arthrosc. 2022 Jan 31;30(7):2352–7. doi: 10.1007/s00167-022-06889-5 (PMC9206617; doi:10.1007/s00167-022-06889-5)
Supplement: Supplementary file 1 — Supplementary file1 (DOCX 25 KB) [file 167_2022_6889_MOESM1_ESM.docx]

**Supplement 1: Survey including responses from the members of the German Knee Society.^a^**

| **Q1. In welchem Land sind Sie als Kniechirurg tätig?** (n = 152)   1. Deutschland (**89.5%**) 2. Österreich (**4.0%**) 3. Schweiz (**5.3%**) 4. Anderes (**1.3%**) |
| --- |
| **Q2. In welchem klinischen Setting sind Sie tätig?** (n = 152)   1. Universitätsklinikum (**15.8%**) 2. Privatklinikum (**19.7%**) 3. Peripheres Klinikum (**40.1%**) 4. Praxisklinik (**17.8%**) 5. Anderes (**6.6%**) |
| **Q3. Sind Sie zertifizierter Kniechirurg? Wenn ja, vor wie vielen Jahren haben Sie die DKG Zertifizierung „Kniechirurg“ erhalten?** (n = 152)   1. < 1 Jahr (**12.5%**) 2. 1-3 Jahre (**17.8%**) 3. 3-5 Jahre (**15.1%**) 4. > 5 Jahre (**11.8%**) 5. Keine Zertifizierung (**42.8%**) |
| **Q4. In welchem Bereich der Kniechirurgie sind Sie überwiegend tätig?** (n = 152)   1. Endoprothetik (**27.0%**) 2. Sportorthopädie (**63.8%**) 3. Traumatologie (**9.2%**) 4. Anderes (**0%**) |
| **Q5. Wie viele meniskuschirurgische Eingriffe führen Sie jährlich durch?** (n = 152)   1. < 30 (**8.6%**) 2. 30-50 (**13.8%**) 3. 50-100 (**23.0%**) 4. 100-200 (**26.3%**) 5. > 200 (**28.3%**) |
| **Q6. Sehen Sie einen klinischen Bedarf zur Meniskus Allograft Transplantation in der Bundesrepublik Deutschland?** (n = 152)   1. Ja (**91.5%**) 2. Nein (**8.5%**) |
| **Q7. Bei wie vielen Patientinnen und Patienten sehen Sie jährlich die Indikation zur Meniskus Allograft Transplantation?** (n = 152)   1. < 5 (**40.8%**) 2. 5-10 (**39.5%**) 3. 10-20 (**12.5%**) 4. > 20 (**7.2%**) |
| **Q8^b^. Was sind für Sie die ausschlaggebendsten Kriterien zur Indikationsstellung einer Meniskus Allograft Transplantation? (mehrere Antworten möglich)?** (n = 152)   1. Patientenalter (**83.6%**) 2. Funktioneller / sportlicher Anspruch (**43.4%**) 3. Isolierte laterale Meniskusinsuffizienz mit begleitendem Postmeniskektomie Syndrom (**79.6%**) 4. Isolierte mediale Meniskusinsuffizienz mit begleitendem Postmeniskektomie Syndrom (**71.7%**) 5. Meniskusinsuffizienz bei begleitender ligamentärer Instabilität (**24.3%**) 6. Meniskusinsuffizienz bei begleitendem koronaren Malalignment (Varus/Valgus) (**21.7%**) |
| **Q9^b^. Welche Therapiemethoden kommen für Sie als Alternativen zur Meniskus Allograft Transplantation im Falle eines Postmeniskektomie-Syndroms in Frage? (mehrere Antworten möglich)?** (n = 152)   1. Keine spezifische Therapie (**2.6%**) 2. Konservative Therapie bestehend aus physiotherapeutischen und physikalischen Behandlungen (**55.9%**) 3. Arthroskopisches Debridement (**13.8%**) 4. Meniskusimplantat (z.B. NUsurface®, CMI, Actifit®) (**46.1%**) 5. Beinachsenkorrektur (**84.9%**) 6. Unikompartimenteller Kniegelenkersatz (**47.4%**) |
| **Q10^b^. Worin sehen Sie die größten Hürden in der Durchführung einer Meniskus Allograft Transplantation in der Bundesrepublik Deutschland? (mehrere Antworten möglich)?** (n = 152)   1. Technischer und operativer Anspruch (**19.7%**) 2. Patientenrekrutierung (**7.2%**) 3. Rechtliche / gesetzliche Situation (**77.6%**) 4. Implantatverfügbarkeit (**76.3%**) 5. Kostenübernahme (**82.9%**) |
| **Q11^b^. Worin sehen Sie den größten Handlungsbedarf in der Meniskus Allograft Transplantation in der Bundesrepublik Deutschland? (mehrere Antworten möglich)?** (n = 152)   1. Gesetzgebung zur Verwendung humanen Spendergewebes (**80.9%**) 2. Aufbereitung der Spendermenisken (**40.1%**) 3. Kostenübernahme durch Krankenkassen (**90.8%**) |
| **Q12^b^. Wie könnte die Deutsche Kniegesellschaft e.V. dazu beitragen für Sie die Anwendung der Meniskus Allograft Transplantation in der klinischen Praxis zukünftig zu vereinfachen/ermöglichen? (mehrere Antworten möglich)?** (n = 152)   1. Erstellung einer Leitlinie „Wie bekomme ich einen Spendermeniskus für meine(n) Patientin/Patienten“ (**75.7%**) 2. Informationsbroschüre für Patientinnen und Patienten zur Indikationsstellung, Durchführung, Rehabilitation, sowie ethischen Aspekten der Meniskus Allograft Transplantation (**48.7%**) 3. Zertifizierte Veranstaltung (Theorie + Workshop) zum Thema „Meniskus Allograft Transplantation“ (**57.9%**) 4. Umfassende Zusammenfassung sämtlicher Literatur (Indikationsstellungen, technische Aspekte, klinische Ergebnisse) im Zuge einer Sonderausgabe des Kniejournals „Meniskus Allograft Transplantation“ (**71.7%**) |
| **Q13. Haben Sie schon einmal eine Meniskus Allograft Transplantation durchgeführt?** (n = 152)   1. Ja (**35.5%**) 2. Nein (**64.5%**) |
| **Q14. Wie viele Meniskus Allograft Transplantationen haben Sie bereits durchgeführt?** (n = 53)   1. < 5 (**64.2%**) 2. 5-10 (**13.2%**) 3. 10-20 (**7.6%**) 4. 20-50 (**11.3%**) 5. 50-100 (**1.9%**) 6. > 100 (**1.9%**) |
| **Q15. Von wo beziehen Sie ihre Spendermenisken?** (n = 53)   1. Deutsches Institut für Zell- und Gewebeersatz gGmbH (DIZG) (**58.5%**) 2. Hauseigene Gewebebank (**5.7%**) 3. Anderes (**35.9%**) |
| **Q16. Welchen Typ an Spendermenisken verwenden Sie?** (n = 53)   1. Fresh-Frozen Spendermenisken (**56.6%**) 2. Kryopreservierte Spendermenisken (**7.6%**) 3. Lyophilisierte Spendermenisken (**0.0%**) 4. Peressigsäure-Ethanol sterilisierte Spendermenisken (**35.9%**) 5. Anderes (**0.0%**) |
| **Q17. Welche Operative Technik verwenden Sie für die Meniskus Allograft Transplantation?** (n = 53)   1. Komplett arthroskopisch (**54.7%**) 2. Komplett offen (**3.8%**) 3. Arthroskopisch und offen kombiniert (**41.5%**) |
| **Q18. Welche Fixationstechnik der Spendermeniskuswurzeln verwenden Sie für eine mediale Meniskus Allograft Transplantation??** (n = 53)   1. Knochenblockfixation (**22.6%**) 2. Reine Weichteilfixation (**73.6%**) 3. Andere (**3.8%**) |
| **Q19. Welche Fixationstechnik der Spendermeniskuswurzeln verwenden Sie für eine laterale Meniskus Allograft Transplantation??** (n = 53)   1. Knochenblockfixation (**24.5%**) 2. Reine Weichteilfixation (**69.8%**) 3. Andere (**5.7%**) |

^a^ ”n” indicates the number of respondents per question. The percentages in parentheses indicate the frequency of the corresponding response among the respondents; ^b^Since more than one answer could be selected, the cumulative percentage may exceed 100%.
